# The Totalising Nature of Secure and Forensic Mental Health Services in England and Wales

**DOI:** 10.3389/fpsyt.2021.789089

**Published:** 2021-11-08

**Authors:** Sarah Markham

**Affiliations:** Department of Biostatistics and Health Informatics, Institute of Psychiatry, Psychology and Neuroscience, King's College London, London, United Kingdom

**Keywords:** forensic—psychiatric practise, ethics—institutional, ethics—clinical, risk, rights activism

## Abstract

This evidence-based opinion piece explores the totalising risk averse nature of secure and forensic mental health services and associated iatrogenic harms in England and Wales. Drawing on the research literature I consider the various influences, both external and internal which impact on the provision of such services and how both the therapeutic alliance and recovery potential for patients may be improved. Especial attention is paid to the deployment of restrictive practise, practitioner attitudes, the potential for non-thinking, and how these may impact on decision-making and the care and treatment of mentally disordered offenders.

## Mentally Disordered Offenders

Patients detained under Part III of the Mental Health Act (1) in England and Wales are required by law to receive specialist care because their mental disorder is perceived as posing a risk of harm to themselves and to the community (2). Secure and forensic mental health services are provided for such patients (3). Risk can manifest at individual, interpersonal, organisational, and community levels (4, 5). Adverse incidents, some having extreme consequences can and do present in secure and forensic mental health settings. Evidence-based understanding of causal factors, authoritative and procedurally just boundary setting, consistent care, treatment, and proportionate monitoring are required to maintain therapeutic efficacy (6).

The care and treatment of mentally disordered offenders involves balancing the therapeutic role with managing perceived risk and maintaining safety and security (7). However, in practise secure and forensic settings place an overriding emphasis on physical and procedural security; ways of working with and treating patients that are viewed as permissible and even necessary, given the stereotypes associated with mentally disordered patients. This can lead to administratively and legislatively driven disregard for patient well-being and even harm. It is recognised that disproportionate risk aversion can lead to patients being deprived of the opportunities they need to progress in their recovery (8).

Within forensic clinical practise risk tends to be treated as an objective reality that can be rationally managed *via* the deployment of expert knowledge and authority. However, early modern anthropological research reified that the way in which risk is perceived and responded to, is determined by social values and institutions rather than evidence-based thinking (9). Risk rather than being a neutral, objective concept is infused with values and beliefs that can exert a significant normalising influence and ultimately determine what is an isn't to be considered as a risk (9).

The concept of risk provides the “raison d'etre for the structure” and operation of secure mental health systems, directing every aspect of the care and treatment of mentally disordered offenders from admission to discharge and beyond (10, p.12). Without the notion of risk and beliefs regarding its assessment and management, these hospitals would not exist. Forensic mental health care spans both criminal justice and healthcare systems and as such is subject to the political, cultural, legal, and economic factors influencing these contexts.

It is recognised that secure and forensic mental health settings can be highly restrictive, coercive, and risk-averse (10, 11). The dominant discourses of modern forensic psychiatry are constituted by reductively simplistic conceptions of the causation of violence. The stigmatisation, lack of rigour in maintaining detention under the MHA (1) and effectively unchecked discretion of the Ministry of Justice (MoJ) in the United Kingdom (UK) regarding the recall to a secure hospital of patients under Section 41 (S41) of the MHA (1) are manifestations of the means by which a modern government and society seek to assuage their sense of ontological security in the face of offences committed by those with a diagnosis of mental disorder.

Forensic psychiatry can be framed as operationalising a system of social control in which individuals with the mentally disordered offender label are stratified according to the risk they are perceived to present to others in high, medium and low secure mental health settings (12). In these settings, treatment and care are delivered within a coercive framework of imposed assessment and therapy (13, 14). Risk assessment and management subsume all other dimensions of care and treatment. They are multi-dimensional processes relating to physical, procedural, and relational security with the over-arching aim of integrating security with therapeutic goals (15, 16). Perceived risk can dominate every aspect of practise and service provision, leading to a culture of containment developing whereby staff increasingly prioritise perceived safety over recovery and favour the deployment of risk-averse approaches (including seclusion and restraint) rather than using more therapeutic forms of intervention (17, 18).

It can be argued that the focus on risk assessment and management discriminates against those with a diagnosis of mental disorder given the mandatory nature of such practises and associated controls placed on patients (19). There is a significant risk of disproportionate risk aversion and coercion given the perceived implications for professionals of failure to predict what may be unpredictable and consequent apportioning of accountability and blame (20). Where risk assessment and management dominate and pervade the of risk provision of care and treatment, together with patients' autonomy, the potential for inappropriate levels of restriction to be imposed upon individuals will present. For instance research has historically proven that a significant number of forensic patients have been placed at unnecessarily high levels of security (21). It has been “argued with reference to empirical data and literature that the defining characteristics of late modern social control” are manifest within forensic mental health services (10, p. 12).

The assessment and management of risk are considered essential skills for forensic mental health staff, along with the implementation of evidenced based interventions (23). However, the extent to which risk presents on wards may be partially associated with the quality of the interactions between staff and patients (24). For instance, more authoritarian approaches to boundary setting may engender a negative response from patients, whereas using an authoritative manner may promote positive outcomes (25). Anxiety-based, subjective, often unreasoned and unevidenced perceptions of potential risk in the context of legal controls directed by the Ministry of Justice lead to the liberty of patients being curtailed indefinitely by practitioners wary of potential damage to their continuing professional development should a rare but serious event be enacted.

Thus various social and structural control processes can impact upon the implementation of strength- and recovery-based approaches to care and treatment in secure and forensic mental health settings. It is recognised that in secure and forensic mental health settings a culture of containment can present in which staff become increasingly unable to deliver intervention which will aid recovery and instead prioritise unsafe certainty *via* the deployment of restrictive measure, both direct and indirect (17, 18). The actualisation of patient empowerment, autonomy, identity, and connectedness can conflict with and be compromised by the punitive influences of disproportionate risk aversion and other forms of containment and control (22).

## Standards of Practise for Offender Recovery

Standards of practise are authoritative statements that reflect current knowledge and understanding along with the values and priorities for a profession and provide stated expectations of accepted performance (26, 27). Standards allow staff to be held accountable for safe, competent, ethical, and legally defensible practise (16). The Royal College of Nursing (RCN) in the UK has “identified the core competencies and advanced nursing practises for mental health nurses working with” mentally disordered offenders (16, p.173; 28). The core competencies were generic mental health nursing competencies; advanced nursing practises included risk assessment and management, assessment and management of dangerousness, cognitive therapies, behavioural therapies, and social skills training (16, 29).

A literature review identified competence in safety and security, risk assessment and management, management of violence, providing therapy, knowledge of offending and legislation and ethics, report writing, understanding the criminal justice system and “jail craft,” as relevant to forensic nursing, together with desirable personal qualities such as an understanding of public attitudes, an appreciation of control and the secure environment, and the nurse and patient relationship (30).

Tension and the potential for challenge are inherent in the context of the care and treatment of mentally disordered offenders. Policies and protocols concerning physical, procedural, and relational security are rooted in distrust and disregard, and patients' legal status conflicts with notions of voluntary treatment. Hence, the imperative for staff to be capable of making optimal use of interpersonal therapeutic skills (4, 31). Secure recovery (promotion of personal, clinical, functional and social recovery, and desistence) requires knowledge of the criminogenic needs of the patient together with the circumstances, nature, and consequences of their offending behaviour, in addition to their personal, clinical, functional and social needs and priorities (32). Therapeutic relationships and ward ambience can serve to facilitate an understanding of offending and other maladaptive behaviours together with mental disorder and other recovery needs (32–34).

## The Carceral State and Secure and Forensic Mental Health Care

Secure and forensic mental health services ostensibly aim to balance care and treatment with custodial objectives and function. However, given the totalising reality of forensic mental health settings, carcerality permeates every aspect of the provision of secure care, as confirmed by the literature describing secure hospitals as dangerous, punitive, and controlling (10). This carcerality is visibly manifest in the physical security on which such services are based and operate, and acts to confound attempts to introduce more trauma-informed ways of working with mentally disordered offenders (10). “The punitive and custodial nature of secure environments may also be mediated by stigmatising and judgmental staff attitudes. In one study staff are reported as stating of patients that ‘they should be having a miserable time. That's not a therapeutic attitude I know, and it doesn't really work very well but I do feel it from time to time'” (10, p.7).

It is the alleged or offending behaviour that differentiates secure and forensic mental health patients. Attitudes regarding mental disorder and offending behaviour are impacted by fear, ignorance, misinformation, and at times sensationalist media coverage. Secure and forensic mental health patients can “evoke feelings of disgust, repulsion and fear” and leave staff feeling unskilled and fearful of their own safety (16).

Patients experience punitiveness daily *via* the enactment of protocols; blanket restrictions and other rules and regulations (22). The spectre of presumed public opinion and the fear of condemnation from the popular press haunts secure and forensic mental health settings and dictates and sustains a philosophy of stigmatisation and oppression.

## Collaborative Risk Assessment and Management

Risk assessment is a mandatory component of care planning and a constant concern for staff with significant consequences for liberty of patients (35). The risk patients present to others though less present than risk to self, has greater salience in both legislation and practise, with greater perceived negative outcomes for both staff and mental health providers (36, 37). Risk assessment policies and practises are developed and implemented within this wider context of concern about possible adverse effects for accessors and the organisations who employ them should they fail to identify and guard against a rare but serious event occurring. There is more emphasis and resource placed on and deployed in mitigating against the incidence of high profile but low probability harms such as patient homicide than the low profile high probability harms sustained by the patient body such as adverse reactions to medication and associated physical health effects, including higher rates of morbidity and mortality, which are seemingly accepted without concern (38).

Risk assessment is a contested area of mental health care, especially in the context of forensic psychiatry. The predictive accuracy of risk assessment in mental health care is sub-optimal; even the best performing actuarial tools perform at a level which is substantially below what is deemed acceptable in other branches of healthcare (37, 39). Reviews have consistently recommended that risk assessment tools and associated scales are not used for routine clinical practise and emphasise the need for a more personalised focus on the individual patient (40).

The weight placed on and the enduring nature of the influence of risk assessments should not be underestimated, yet those subject to them often have little involvement in the process and related decision-making. Research has indicated that patients and staff have contrasting and at times competing priorities in relation to risk assessment and management (36). Patients view risk as a staff driven priority that may lead to restriction and loss of liberty (36). Staff claims of involving people in the care planning process do not extend to risk assessment and management processes (36).

Staff attribute risk to originating in the “patient rather than social or environmental factors, are risk averse and prioritise the procedural aspects of risk assessment” (36, p.471). Risk assessment practise operates as a form of fiction in which poor predictive ability and subjective fear of adverse consequences are accepted in the interests of presumed normative certainty (36). Contrary to best practise guidance staff may inevitably default to the false security of unsafe certainty regardless of the costs to both individual patients and tax-payers of unnecessary levels of supervision and monitoring including overlong lengths of stay (41). As a consequence, risk adverse options are preferred by staff and patients discouraged from taking advantage of opportunities for ordinary risks thereby hindering the development and maintenance of their personal recovery (36).

While risk assessment and management processes focus on risk of self-harm, suicide and harm to others, the risk of iatrogenic harm, i.e., harm associated with the provision of care and treatment such as adverse reactions due to psychotropic medicine is invariably neglected (42). Other risks to which patients may be vulnerable include discrimination, stigma and verbal, and physical aggression (43). Patients may find it difficult to assert their rights and experience a profound sense of powerlessness in the face of bureaucracy and uncaring staff (44).

Collaborative risk assessment and management have been recommended in health policy for over a decade in the UK (45). However, there is evidence that the extent to which patients are involved in risk assessment is suboptimal (46, 47). Patients are often not aware of the content of their risk assessments let alone included in their development (48). There appears to be a discrepancy between the beliefs staff articulate and their statements about being open to collaborative risk assessment and their practise (14). There is evidence that patients are often not aware of risk assessments being done (49) and that assessments place significantly more emphasis on individual risk factors than structural, social or interactional issues (36). By not allowing patients opportunities to be meaningfully involved in risk assessment and management and develop their own understanding and knowledge regarding risk, staff are culpable of epistemic injustice (50).

Patients may disagree with the contents of their risk assessments, but feeling they have little influence, may perceive that there is no value in contesting them (48). Patients may also seek to minimise their risk status through compliance with staff's views (51). They may believe that contesting the content of a risk assessment may be interpreted as a lack of insight on their part and thus an indicator of risk in itself (52). It is important to be able to understand how patients experience the processes of violence risk assessment and management in order to optimise engagement and meaningful collaboration.

Collaborative risk assessment and management have been recommended for over a decade (53). This involves a joint decision-making process between patients and staff with the patient involved in each part of the process including the identification of risks and appropriate level of support they need to mitigate risk (13, 14). Collaborative risk assessment can become the first step towards patients becoming accountable for and managing their own risk. The collaborative process can also enhance patients' understandings of why certain interventions are viewed as required and support them to feel empowered (54). Other positive consequences of collaboration include ensuring relevant information is not missed, the identification and provision of insight on warning signs which may not be obvious to staff (13, 55). Collaborative risk assessment and management may also lead to patients taking increased accountability for their own recovery (56), and providing information on their internal mental states which are associated with risk (57). However, the research which has been conducted indicates that the extent to which collaborative risk assessment is occurring may be suboptimal (46, 47). However, evidence of the value of collaborative care in evaluating risk in secure and forensic settings does exist, and remains a possible means of improving forensic care (58).

## Barriers to Authentic Therapeutic Relationships and Patient Recovery

The recovery paradigm has become the mandated model for secure and forensic mental health services over the last decade (59). The recovery model is a strengths-based approach which involves clinicians supporting patients to lead satisfying and meaningful lives in the context of their mental disorder (60). The Secure Recovery model focuses on the role of therapeutic relationships, active participation in recovery and developing a sense of responsibility and self-agency (32, 61). It is recognised that the therapeutic alliance can act as a vehicle to keep patients safe and manage their needs and risks (62). However, secure and forensic mental health services place favour the concept of the managed patient rather than having regard for patient agency or autonomy. Mental health legislation empowers staff and disempowers patients. Staff may deploy statutory powers in the context of perceptions of risk, whereas patients may lose their liberty and be compelled to accept treatments that they would not otherwise choose.

It is recognised that mentally disordered offenders form a marginalised social group predominantly due to the dual stigma associated with both the mentally unwell and criminal identities (10, 63). Attitudes towards mental disorder and offending behaviour are shaped by ignorance, fear, misinformation, and sensationalist representation in the media. Patients have expressed the concern that such stigma will negatively impact upon their recovery (10). Such stigma is enduring and likely to remain with mentally disordered offenders after discharge and affect their reintegration into the community, influencing housing, occupational, and social opportunities (10). It can act as a barrier to opportunities to find work, and other means of social integration and well-being.

It has been found that staff in forensic contexts had difficulty in articulating exactly what it is that they did that might be therapeutic (64). Examination of staff case file entries in a secure and forensic mental health unit failed to confirm the nurses' contention that their practise was comprehensive and therapeutic (65). Negative appraisals from others together with the internalised impacts on self-concept of the mentally disordered offender identity and conditions of existence can present significant barriers to personal recovery. An inability to think on the part of staff, i.e., to fully empathise, consider and understand a patient in a given situation, coupled with subjective self-protective anxieties can lead to the potential for significant iatrogenic harm. Understanding, support, advocacy and education are required to combat stigma and discrimination within and outside of secure and forensic mental health services (66).

Staff may, on a daily basis, be involved in making decisions that necessitate conflicts between multiple ethical, legal and societal values (67). This raises the potential for moral injury and concerns regarding the psycho-emotional aspects of decision making, such as feelings of regret and shame (68, 69). Staff may feel compromised due to the seeming contradiction of providing care and treatment while protecting the public. The phenomenon of accepted fictions can present in that staff may recognise that the basis for certain approaches may be predominantly administrative and have no scientific validity (39). Staff may also prefer to avoid potentially problematic conversations regarding risk and offending behaviour for fear of this damaging the therapeutic relationship (46, 70). The process of developing therapeutic alliances, and experiencing trust or even rapport can also be problematic due to the restrictive nature of secure and forensic mental health services. The individuals who come to be mentally disordered offenders may also have been exposed to neglectful or cruel experiences in early life (58). Trauma can be an integral part of the experience of being a mentally disordered offender; trauma related to committing an index offence, detention (isolation from the community and personal contacts), coercion in secure settings, and the impact of the totalising nature of the secure environment. Legal status and enduring mental illness can result in significantly long lengths of stay leading to the risk of loss of hope and institutionalisation.

Staff have also reported concerns for their own security (71). Aggression when it presents can have multiple adverse consequences for patients, staff, ward atmosphere and operation (72, 73). Patients who express aggression may be met with restrictive interventions such as sedation, seclusion or restraint (73). A reliance on relational as opposed to procedural or physical modes of security may require staff to challenge and overcome paternalistic perspectives and associated assumptions regarding risk.

Patients have articulated frustration regarding the dominance of the staff's views together with their sense of helplessness and inability to change the status quo. Having to ask permission to meet basic needs can result in patients feeling disempowered and lacking agency. Patients perceive relationships with staff as distorted due to the significant power differential which exist. Patients may have a strong desire for change, compounded by perceptions of powerlessness. Compliant behaviour may seem the only practicable way to progress leading to symptoms and concerns being masked or downplayed, and patients regulating what they communicate to staff. This can lead to increased levels of frustration and impede the recovery process. Even when well-managed by the patient, passivity and compliance rather than active engagement will likely lead to sub-optimal outcomes. Thus, meaningful and effective therapeutic relationships can be difficult to initiate and sustain in secure and forensic mental health settings. The barriers mentally disordered offenders face in negotiating and achieving recovery should not be under-estimated. However, national and local quality improvement networks such as the Royal College of Psychiatrist's Quality Network for Forensic Mental Health Services which organises peer reviews of medium and low secure and forensic hospitals with a view to increasing standards of care for patients and sharing good practise have demonstrated success in improving the quality of patient care and experience (74).

## The Potential for Abuse of Power—Staff Attitudes and Accountability

The morality of decisions can become dependent on the context; who and what is being prioritised; perhaps what is best for the service, ward or individual practitioner as opposed to the patients. Disregard and harm on the grounds of exceptionalism, predicated on the dubious notion that practitioners are innately superior and shouldn't be held to the same standards when dealing with individuals whose human rights are qualified due to past offending behaviour are insidious and yet potentially pervasive wrongs. Without internalised or externalised structures of personal or moral responsibility, accountability and monitoring, the nature and extent of the disregard enacted upon patients may become unlimited. Practitioners who routinely engage in harm, but may consistently claim that on the contrary, that they are engaged in good practise need robust supervision and monitoring. A relationally secure See Think Act framework for professional practise, supervision, vigilance, and ultimately whistle-blowing would potentially be of great benefit to services and patients (75).

The greater the power differential between staff and patients, the greater the potential for abusive staff behaviour (76). Milgram's obedience studies led to the development of the concept of a drone like “agentic state” in which individuals suspend their capacity to make informed moral judgments and relinquish responsibility for what they do to those in authority (77). Individuals may abdicate their moral agency by acting primarily to mitigate their subjective, self-protective anxieties, regardless of the harm it may cause to others. “*You have to protect your back*.” Zimbardo suggested that such a sense of obligation and duty is not necessarily dependent on the presence of strong authority figures, but can be due to individuals conforming to what they believe is expected of them as a group member. Whether, staff follow the policies and practises set by those in authority or prioritise individual patient need and well-being can depend upon the extent to which they perceive themselves to share social identification with either group (78, 79).

The restrictive ethos of secure and forensic settings can compromise a patient's individuality in various ways, leading to an overall sense of powerlessness (80). In some circumstances, for example a secure and forensic mental health ward with a high incidence of violence, authoritarian leadership might provide relief and protection against the environmental uncertainties (81). Workers may then displace the responsibility for their actions onto their superiors: “*It's not up to me, I don't make the decisions, I just do what I'm told to do*.” In such situations staff may perceive it to be a virtue to over-restrict patients; that they deserve it, for the violence they have committed and the potential for further violence that they are perceived to possess.

An inability of practitioners to identify with their patients can lead them to be unaware of the potential gulf in human suffering that separates them (the oppressors) from their patients (the oppressed). Incapable of thinking from the perspective of one labelled as the alien inferior and innately unreliable other (the mentally disordered offender) practitioners may by default fail to take account of or priorities their patients' self-articulated needs.

Barriers to the proportionate deployment of relational as opposed to more restrictive and oppressive forms of security and ways of working with and relating to patients could include negative staff attitudes, competing organisational priorities, and organisational inertia. The work of staff can tend to be more functional and task oriented, rather than relationally focused (82). Research has found that staff may have difficulty in articulating exactly what it is that they do that might be therapeutic (64). Nurses might distance themselves from patients in order to cope with conflict and other relational difficulties (83). It can necessitate resilience and to care for patients who exhibit such demanding presentations (84).

Staff in secure and forensic settings tend to attribute conflict with patients, including presentations of violence and aggression, either to mental illness or other deeply ingrained aspects of patients' personalities (85, 86). This is consistent with the broader literature that indicates mental health staff generally tend to attribute patient aggression to internal factors, such as patient psychopathology, more readily than environmental or situational factors including communication between the patient, staff member, and other patients (87). A study found that staff reframe restrictive practise as acts of compassion and necessary means of managing risk, thereby reducing feelings of unease derived from constantly acting against the will and wishes of their patients (88).

The beliefs that relational security is of secondary importance to physical and procedural security, or that it already exists in practise or presents by default when situations arise that aren't explicitly covered by other forms of security, can present further attitudinal barriers to the consistent and effective implementation of relational security. Other barriers to proportionate emphasis on relational security include heightened acuity, demands on staffing resources, and criticism of the process of implementation (86).

Other unhelpful staff attitudes and behaviours include being judgemental, confrontational, and over-reacting (89). Staff need to be risk aware and risk assessment competent whilst being able to confidently hold onto uncertainty. It is important to balance security and safety with ensuring equity of care whereby the forensic mental health patient is treated the same as any other mental health patient. The ability of staff to recognise and acknowledge their feelings towards patients' behaviours can be important in determining how staff exercise relational security (25). Policy, procedures and the quality and consistency of staff supervision and reflective practise also impact hospital and relational culture, and ultimately staff behaviour and relational security (25).

Concerns have also been expressed that relational security; developing a knowledge and understanding of patients' inner and outer worlds may be misused by staff as a means of controlling patients rather than to promote meaningful recovery (90, 91). Given the length of stays in secure settings, and thus the long periods of time patients spend in the company of staff, it is understandable that how staff treat individuals can impact significantly on their self-concept, self-esteem, and potential for sustained recovery. I would suggest that evidence-based initiatives to improve the quality of relational security as it is deployed within secure and forensic mental health settings would be of value to both patients and staff.

## Reducing Coercion and Restrictive Practises

It is recognised that in secure and forensic mental health settings a culture of containment can present in which staff become increasingly unable to deliver interventions which will aid recovery and instead prioritise unsafe certainty *via* the deployment of restrictive measures, both direct and indirect (17, 18).

Restrictive practise refers to the broader context of confinement, including the ward environment, dynamics, atmosphere, and routines, in addition to restrictive interventions. A distinction may be drawn between direct coercion (e.g., rapid tranquilisation, seclusion etc.) indirect coercion (e.g., restrictive rules and regulations, a controlling ward atmosphere, etc.), and informal coercion (which patients may refer to as “pressure”) (92). Restrictive practises can conflict with individuals' attainment of their human rights, for example autonomy, physical integrity, and liberty of choice or movement (93). Research has indicated that the more restrictive the environment and approach to care, the higher the levels of depression and suicidal ideation, hostility, disrespect for patients, and perceived lack of institutional transparency. Lack of autonomy can lead to patients feeling punished and disempowered through having to rely excessively on staff. Restrictive practises can lead to harmful consequences such as physical injury or death, mental health deterioration (including the onset of post-traumatic stress disorder), and increased length of detention (94).

The experiences of restrictive practises can be enacted *via* means which are in sensitive, distal, and bureaucratic, as well as visible, routine and coercive (59). A concept analysis of restrictiveness in secure and forensic mental health settings identified two key factors; paternalistic attitudes towards care and treatment, and the dominance of the concept of risk assessment and management (10). In addition to formal forms of coercion, patients may experience implicit coercion in the form of pressure to achieve therapeutic goals in which they have played no part in setting, and which they experience insufficient if any support.

“Yes, the expectations are to achieve goals. And if it doesn't work, they don't ask what the problem is. Instead, it's said, ‘You have to’ instead of communicating with each other about this issue. It is always—how shall I put it? It's defined what we have to do and not talked about what makes it troublesome to achieve it. If goals are not met, there is no support, there is more pressure” (88).

Reducing and eliminating the use of restrictive practises, such as seclusion and restraint, has become both a national and international priority and focus of mental health policy reform (95, 96). In order to reduce the deployment of restrictive practises there have been legislative reforms, and changes in policy and best practise guidelines in the UK (1, 97, 98). The National Collaborating Centre for Mental Health recently implemented the Reducing Restrictive Practise Collaborative, a large-scale initiative that aimed to reduce restrictive practise by 33% across 26 NHS trusts in England (99). However, secure and forensic services continue to report high rates (100) and it has been evidenced that they may form part of a “vicious cycle” in which the psychological perturbation and distress they cause lead to more maladaptive behaviours, and in turn further coercive measures that in turn result in further restrictive practises (101). It has been evidenced that restrictive practises are associated with harms such as anxiety, trauma, disorientation and perceptions of neglect and abuse (92, 102).

Specific focus on secure mental health services is warranted as restrictive practises are often viewed as an integral part of forensic psychiatry but have received limited research attention relative to other areas of psychiatric practise (101, 103). Patients have reported that coercion is applied in a disproportionate way not only in terms of individual measures but to the system as a whole (88). A correlation has been found between disruptive behaviour, violence, and seclusion use in relation to sense of community and ward climate (104).

Proactive approaches should be used to mitigate the potential for harm in a proportionate and personalised manner (105). Unit culture is the core factor in influencing the use of restrictive practises (18). Therefore, the development and maintenance of relationally secure environments can play a key part in minimising restrictive practises. A scoping review of the use of restrictive practises, the consequences of using them and efforts to reduce restrictive practises, in adult secure and forensic mental health settings recommended that the importance of collaborative working (106).

Research has indicated that staff's emotional world can affect the deployment of restrictive measures with higher levels of anger likely to lead to the endorsement of management techniques such as the use of restraint, whereas those who experienced higher levels of guilt were less likely to sanction the use of seclusion (107). A study found that staff reframe restrictive practise by describing interventions as acts of compassion and as necessary means of managing risk, thereby reducing feelings of unease derived from constantly acting against the will and wishes of their patients (88). The manner in which staff process and understand their actions has an impact on their emotional reactions to the ways in which they interact with patients. This indicates a need for regular supervision and reflective practise (108). To mitigate the barriers to reducing restrictive practises posed by staff perceptions and attitudes, the introduction of staff training which utilises a co-creation approach has been shown to be beneficial (109, 110).

A caring, proportionate and authoritative, rather than authoritarian, boundary-setting style potentiates positive outcomes (25). Asking rather than telling patients what to do has proven more effective in managing behaviour (25). “Setting limits in an authoritarian as opposed to an authoritative manner can be experienced by patients as aggressive and disrespectful”, and as a result, may increase rather than decrease the risk of uncooperative and other forms of maladaptive behaviour (25, p.157).

Patients understand and accept that boundaries need to be set and are thankful when limits are set on other patients' behaviours, as this is perceived to protect their own wellbeing and the therapeutic milieu (25). Patients also acknowledge the need for boundaries in therapeutic relationships (111) including the degree of affective involvement (24). One study found that the efficacy of boundary setting was optimised when boundaries were set firmly, but empathetically *via* mutual agreement, and consistently applied among all patients (112). A caring, proportionate and authoritative, rather than authoritarian, boundary-setting style can therefore potentiate positive outcomes (25).

## Professional Judgement and Decision Making in Secure and Forensic Services

Patients have expressed the need for an ethical authority that monitors forensic psychiatrists and secure and forensic mental health services (88). This demonstrates the existence of a perceived need for protection from arbitrariness. In order to understand decision-making we need to understand both the individual decision maker and the context in which they make decisions (113). The fear of making “mistakes” may hinder good practise (114). “Psychological safety is the perception that expressing ideas, opinions and reporting concerns, or mistakes won't lead to humiliation or punishment” (115, 3:48). A sense of psychological safety is necessary if staff are to feel confident in taking proportionate risks and innovation. “The three most powerful behaviours that foster psychological safety are being available and approachable, explicitly inviting input and feedback, and modelling openness and fallibility” (116).

Improving the quality of decision making in secure and forensic mental health settings also requires practicable knowledge of the efficacy of interventions, skills in managing decision processes; and a knowledge base for reflective practise (115). There is a real need for staff to be able to relate their perceptions and understandings of risk to practicable modalities of proportionate preventive intervention and organisational risk management systems. Within this clear delineation is required of the communication and decision-making processes associated with risk assessment together with knowledge of how the benefits and costs of associated actions may impact on patients. The effective assessment and use of both strengths and risk factors may protect against disproportionate risk aversion. The development of evidence-based frameworks which allow the modelling and delineation of what constitutes reasoned, reasonable decision-making in the context of perceived risk would significantly benefit patients.

## Human Rights Advocacy

It has been posited that grounding arguments for strength- and recovery-based principles in the heuristic framework of human rights can offer a set of common values to stimulate reform in forensic mental healthcare (10). Article 8 of the European Convention on Human Rights (ECHR) and Fundamental Freedoms (right to respect for private and family life, home and correspondence) protects individuals' “physical, psychological, or moral integrity,” “privacy,” and “identity and autonomy” (10, 119). Article 8 presents a clearly defined and robust framework to support emphasis on more recovery oriented ways of working. Referring to the ECHR (119), Tomlin and Jordan comment that qualification of these rights is “permitted under but only where any restriction is in accordance with national law and is necessary in a democratic society in the interests of national security, public safety or the economic well-being of the country, for the prevention of disorder or crime, for the protection of health or morals, or for the protection of the rights and freedoms of others.” (10, p.13).

It has been argued that the substantive rights contained within Art. 8 ECHR (117) are aligned with the essential components of strength- and recovery-based approaches (22). Therefore, the imposition of barriers to the enactment of these principles can be contested within a cogent human rights framework. However, such approaches can be rendered ineffective by the various influences and issues they are in theory supposed to mitigate. Nevertheless, any efforts to educate staff and strengthen the application of human rights legislation in secure and forensic mental health settings has the potential to be of value to patients.

## Acknowledging the Needs and Humanity of Mentally Disorder Offenders

The contrast between the passive reality of being a restricted and managed patient in a secure and forensic mental health setting, and the aspiration of being an autonomous, reflexive, and active consumer of mental health care can create both frustration and despair. Agency is denied in the context of the deployment of mental health legislation to restrict liberty and impose treatments. Patients may have minimal access to the community regardless of their length of stay, be denied access to all but a limited number of their possessions and prevented from forming intimate relationships (59).

Secure and forensic mental health care has been mired in a problematic discourse that frames the forensic context and maladaptive attitudes and behaviours of the patient as impediments to good practise. There is an unjustified yet widely held view that mentally disordered offenders lack the mental capacity for moral responsibility and accountability (118). The scepticism regarding the capacity for forensic patients to account for past offending behaviour should not be under-estimated (119). An explicit acknowledgement and understanding of how mental health factors mediate the mutative aspects of interventions is required at the individual patient level, taking into account clinical, personal, social and political dimensions together with the organisational factors that are needed to create the necessary and sufficient conditions for a mentally disordered offender to experience meaningful and lasting recovery.

## Conclusion

Regardless of the nature and extent of the potential for psychological and other interventions to effect adaptive change at the individual patient level, detention in totalising institutions can act to compromise the possibility of recovery. Given the potential for disproportionate risk aversion, unjustified qualification of human rights and sub-optimal patient outcomes, there is a real need for the development of theoretical conceptualizations that direct and inform research regarding what constitutes sound professional judgement, decision, and assessment processes, in the context of offender recovery.

Reductively simplistic and pejorative forensic psychiatric discourses frame mentally disordered offenders as innately unreliable, inferior risk entities lacking the grounding of experiential insight. The moral cynicism of managers and practitioners, their belief that everything is permitted for them, may rest on a solid conviction that authority conveys moral and epistemic superiority. Patients may be subject to punishment or punitive attitudes, othering, and multiple associated barriers to re-integration into society. Reform and progress within the provision of secure and forensic mental health services and practise require the deconstruction of the polarised distinction between offenders and non-offenders, as no one is entirely innocent of moral and other transgressions.

It has been suggested that a human rights approach might counter the detrimental effects experienced by patients who exist at the totalising nature of secure and forensic services. In order to allow for the possibility of positive change and self-restoration, it is necessary to validate the humanity and experiences of patients. Without this there may be no inner healing and outward behaviour change may be neither authentic nor sustainable.

To recognise that (permanent) positive change and progression are possible and can be actualised, are crucial for the recovery of mentally disordered offenders. Within this proportionate patient involvement in decisions and actions relating to their own care and well-being can act as a vehicle for secure recovery and the transition of the mentally disordered offender into an accountable, responsible and responsive member of the community.

## Data Availability Statement

The original contributions presented in the study are included in the article/supplementary material, further inquiries can be directed to the corresponding author/s.

## Author Contributions

The author confirms being the sole contributor of this work and has approved it for publication.

## Conflict of Interest

The author declares that the research was conducted in the absence of any commercial or financial relationships that could be construed as a potential conflict of interest.

## Publisher's Note

All claims expressed in this article are solely those of the authors and do not necessarily represent those of their affiliated organizations, or those of the publisher, the editors and the reviewers. Any product that may be evaluated in this article, or claim that may be made by its manufacturer, is not guaranteed or endorsed by the publisher.
