# Corrigendum: The totalising nature of secure and forensic mental health services in England and Wales

**DOI:** 10.3389/fpsyt.2024.1434728

**Published:** 2024-07-19

**Authors:** Sarah Markham

**Affiliations:** Department of Biostatistics and Health Informatics, Institute of Psychiatry, Psychology and Neuroscience, King’s College London, London, United Kingdom

**Keywords:** forensic—psychiatric practise, ethics—institutional, ethics—clinical, risk, rights activism

In the published article there were several instances of text overlap without appropriate acknowledgement of the original source, as follows:

In the published article, there was an error in **Mentally Disordered Offenders**, Paragraph 4. This sentence previously stated:

“The concept of risk provides the raison d’etre for the structure and operation of secure mental health systems, directing every aspect of the care and treatment of mentally disordered offenders from admission to discharge and beyond.”

The corrected sentence appears below:

“The concept of risk provides the “raison d’etre for the structure” and operation of secure mental health systems, directing every aspect of the care and treatment of mentally disordered offenders from admission to discharge and beyond (10, p.12).”

In the published article, there was an error in **Mentally Disordered Offenders**, Paragraph 7. This sentence previously stated:

“It has been argued with reference to empirical data and literature that the defining characteristics of late modern social control are manifest within forensic mental health services (22).”

The corrected sentence appears below:

“It has been “argued with reference to empirical data and literature that the defining characteristics of late modern social control” are manifest within forensic mental health services (10, p. 12).”

In the published article, there was an error in **Standards of Practise for Offender Recovery**, Paragraph 1. This sentence previously stated:

“The Royal College of Nursing (RCN) in the UK has identified the core competencies and advanced nursing practises for mental health nurses working with mentally disordered offenders (28). The core competencies were generic mental health nursing competencies; advanced nursing practises included risk assessment and management, assessment and management of dangerousness, cognitive therapies, behavioural therapies, and social skills training (29).”

The corrected sentence appears below:

“The Royal College of Nursing (RCN) in the UK has “identified the core competencies and advanced nursing practises for mental health nurses working with” mentally disordered offenders (16, p.173; 28). The core competencies were generic mental health nursing competencies; advanced nursing practises included risk assessment and management, assessment and management of dangerousness, cognitive therapies, behavioural therapies, and social skills training (16, 29).”

In the published article, there was an error in **The Carceral State and Secure and Forensic Mental Health Care**, Paragraph 1. This sentence previously stated:

“Secure and forensic mental health services ostensibly aim to balance care and treatment with custodial objectives and function. However, given the totalising reality of forensic mental health settings, carcerality permeates every aspect of the provision of secure care, as confirmed by the literature describing secure hospitals as dangerous, punitive, and controlling (35). This carcerality is visibly manifest in the physical security on which such services are based and operate, and acts to confound attempts to introduce more trauma-informed ways of working with mentally disordered offenders (36).”

The corrected sentence appears below:

“Secure and forensic mental health services ostensibly aim to balance care and treatment with custodial objectives and function. However, given the totalising reality of forensic mental health settings, carcerality permeates every aspect of the provision of secure care, as confirmed by the literature describing secure hospitals as dangerous, punitive, and controlling (10). This carcerality is visibly manifest in the physical security on which such services are based and operate, and acts to confound attempts to introduce more trauma-informed ways of working with mentally disordered offenders (10).”

In the published article, there was an error in **The Carceral State and Secure and Forensic Mental Health Care**, Paragraph 1. This sentence previously stated:

“The punitive and custodial nature of secure environments may also be mediated by stigmatising and judgmental staff attitudes. In one study staff are reported as stating of patients that ‘they should be having a miserable time. That’s not a therapeutic attitude I know, and it doesn’t really work very well but I do feel it from time to time’ (37).”

The corrected sentence appears below:

““The punitive and custodial nature of secure environments may also be mediated by stigmatising and judgmental staff attitudes. In one study staff are reported as stating of patients that ‘they should be having a miserable time. That’s not a therapeutic attitude I know, and it doesn’t really work very well but I do feel it from time to time” (10, p.7).”

In the published article there was an error in **Collaborative Risk Assessment and Management**, Paragraph 4. This sentence previously stated:

“Staff attribute risk to originating in the patient rather than social or environmental factors, are risk averse and prioritise the procedural aspects of risk assessment (39).”

The corrected sentence appears below:

“Staff attribute risk to originating in the “patient rather than social or environmental factors, are risk averse and prioritise the procedural aspects of risk assessment” (36, p.471).”

In the published article there was an error in **Collaborative Risk Assessment and Management**, Paragraph 6. This sentence previously stated:

“There is evidence that patients are often not aware of risk assessments being done (52) and that assessments place significantly more emphasis on individual risk factors than structural, social or interactional issues (53).”

The corrected sentence appears below:

“There is evidence that patients are often not aware of risk assessments being done (49) and that assessments place significantly more emphasis on individual risk factors than structural, social or interactional issues (36).”

In the published article there was an error in **Barriers to Authentic Therapeutic Relationships and Patient Recovery**, Paragraph 2. This sentence previously stated:

“It is recognised that mentally disordered offenders form an “othered” and marginalised social group predominantly due to the dual stigma associated with both the mentally unwell and criminal identities (67). Attitudes towards mental disorder and offending behaviour are shaped by ignorance, fear, misinformation, and sensationalist representation in the media. Patients have expressed the concern that such stigma will negatively impact upon their recovery (67). Such stigma is enduring and likely to remain with mentally disordered offenders after discharge and affect their reintegration into the community, influencing housing, occupational, and social opportunities (68).”

The corrected sentence appears below:

“It is recognised that mentally disordered offenders form a marginalised social group predominantly due to the dual stigma associated with both the mentally unwell and criminal identities (10, 63). Attitudes towards mental disorder and offending behaviour are shaped by ignorance, fear, misinformation, and sensationalist representation in the media. Patients have expressed the concern that such stigma will negatively impact upon their recovery (10). Such stigma is enduring and likely to remain with mentally disordered offenders after discharge and affect their reintegration into the community, influencing housing, occupational, and social opportunities (10).”

In the published article there was an error **Reducing Coercion and Restrictive Practises**, Paragraph 7. This sentence previously stated:

“Setting limits in an authoritarian as opposed to an authoritative manner can be experienced by patients as aggressive and disrespectful, and as a result, may increase rather than decrease the risk of uncooperative and other forms of maladaptive behaviour (25).”

The corrected sentence appears below:

““Setting limits in an authoritarian as opposed to an authoritative manner can be experienced by patients as aggressive and disrespectful”, and as a result, may increase rather than decrease the risk of uncooperative and other forms of maladaptive behaviour (25, p.157).”

In the published article there was an error in **Professional Judgement and Decision Making in Secure and Forensic Services**, Paragraph 1. A reference to Edmondson (2014) was missing. This sentence previously stated:

“Psychological safety is the perception that expressing ideas, opinions and reporting concerns, or mistakes won’t lead to humiliation or punishment.”

The corrected sentence appears below:

““Psychological safety is the perception that expressing ideas, opinions and reporting concerns, or mistakes won’t lead to humiliation or punishment” (115, 3:48).”

In the published article there was an error in **Professional Judgement and Decision Making in Secure and Forensic Services**, Paragraph 1. A reference to Conley (2018) was missing. This sentence previously stated:

“The three most powerful behaviours that foster psychological safety are being available and approachable, explicitly inviting input and feedback, and modelling openness and fallibility.”

The corrected sentence appears below:

““The three most powerful behaviours that foster psychological safety are being available and approachable, explicitly inviting input and feedback, and modelling openness and fallibility” (116).”

In the published article there was an error in **Human Rights Advocacy**, Paragraph 1. This sentence previously stated:

“It has been posited that grounding arguments for strength- and recovery-based principles in the heuristic framework of human rights can offer a set of common values to stimulate reform in forensic mental healthcare (22). Article 8 of the European Convention on Human Rights (ECHR) and Fundamental Freedoms (right to respect for private and family life, home and correspondence) protects individuals’ “physical, psychological, or moral integrity,” “privacy,” and “identity and autonomy” (121, 122).”

The corrected sentence appears below:

“It has been posited that grounding arguments for strength- and recovery-based principles in the heuristic framework of human rights can offer a set of common values to stimulate reform in forensic mental healthcare (10). Article 8 of the European Convention on Human Rights (ECHR) and Fundamental Freedoms (right to respect for private and family life, home and correspondence) protects individuals’ “physical, psychological, or moral integrity,” “privacy,” and “identity and autonomy” (10, 119).”

In the published article there was an error **Human Rights Advocacy**, Paragraph 1. This sentence previously stated:

“Qualification of these rights is permitted under the ECHR (122) but only where any restriction is “in accordance with national law and is necessary in a democratic society in the interests of national security, public safety or the economic well-being of the country, for the prevention of disorder or crime, for the protection of health or morals, or for the protection of the rights and freedoms of others.”

The corrected sentence appears below:

“Referring to the ECHR (119), Tomlin and Jordan comment that qualification of these rights is “permitted under but only where any restriction is in accordance with national law and is necessary in a democratic society in the interests of national security, public safety or the economic well-being of the country, for the prevention of disorder or crime, for the protection of health or morals, or for the protection of the rights and freedoms of others.” (10, p.13).”

The author apologizes for these errors and states that this does not change the scientific conclusions of the article in any way. The original article has been updated.
